# SrTiO_3_ Nanocube-Doped Polyaniline Nanocomposites with Enhanced Photocatalytic Degradation of Methylene Blue under Visible Light

**DOI:** 10.3390/polym8020027

**Published:** 2016-02-15

**Authors:** Syed Shahabuddin, Norazilawati Muhamad Sarih, Sharifah Mohamad, Juan Joon Ching

**Affiliations:** 1 Polymer Research Laboratory, Chemistry Department, Faculty of Science, University of Malaya, 50603 Kuala Lumpur, Malaysia; syedshahabuddin@siswa.um.edu.my (S.S.); sharifahm@um.edu.my (S.M.); 2 Nanotechnology & Catalysis Research Centre, University of Malaya, 50603 Kuala Lumpur, Malaysia; jcjuan@um.edu.my

**Keywords:** polyaniline, photocatalyst, nanocomposites, methylene blue, SrTiO_3_ nanocubes

## Abstract

The present study highlights the facile synthesis of polyaniline (PANI)-based nanocomposites doped with SrTiO_3_ nanocubes synthesized via the *in situ* oxidative polymerization technique using ammonium persulfate (APS) as an oxidant in acidic medium for the photocatalytic degradation of methylene blue dye. Field emission scanning electron microscopy (FESEM), transmission electron microscopy (TEM), thermogravimetric analysis (TGA), X-ray diffraction (XRD), UV–Vis spectroscopy, Brunauer–Emmett–Teller analysis (BET) and Fourier transform infrared spectroscopy (FTIR) measurements were used to characterize the prepared nanocomposite photocatalysts. The photocatalytic efficiencies of the photocatalysts were examined by degrading methylene blue (MB) under visible light irradiation. The results showed that the degradation efficiency of the composite photocatalysts that were doped with SrTiO_3_ nanocubes was higher than that of the undoped polyaniline. In this study, the effects of the weight ratio of polyaniline to SrTiO_3_ on the photocatalytic activities were investigated. The results revealed that the nanocomposite P-Sr500 was found to be an optimum photocatalyst, with a 97% degradation efficiency after 90 min of irradiation under solar light.

## 1. Introduction

Presently, one of the most essential issues in pollution control from an environmental and biological point of view is the removal of toxic chemicals from waste water. Due to the deliberate or unintentional discharge of dye effluents into aquatic bodies, the textile industry presents a worldwide pollution problem causing a major impact on the quality of available water resources. Textile treatment and dyeing contributes around 17%–20% of the total industrial water pollution as per the World Bank estimate [1,2]. 3,7-bis(Dimethylamino)-phenothiazin-5-ium chloride, generally known as methylene blue (MB), is a heterocyclic organic dye, which is commercially used for various potential applications, including food additives, plastic, paper, pharmaceutical, textile and leather industries [3,4]. It is an as an odorless, dark-green solid powder, which produces a navy blue solution upon dissolution in water and is an extremely harmful chemical that is primarily used as a dye. The ample amount of dye effluents dumped into the environment may possibly result in extreme oncogenic effects, including vomiting, diarrhea, cyanosis, jaundice, quadriplegia, tissue necrosis, distortion of ecosystems and discomposure in aquatic life, which oblige the effective and ample degradation of MB [5,6,7,8]. Photocatalysis has appeared as one of the most promising techniques amongst the wide variety of approaches to degrade menacing waste materials, specifically organic compounds, to less noxious or less harmful materials, because it provides a simple method of exploiting the energy of either natural sunlight or artificial illumination, such as ultraviolet light, microwaves, *etc*. [9,10,11].

Due to the remarkable photochemical, physicochemical and electrochemical properties of inorganic semiconducting materials, they have found a wide variety of applications in the fields of photoluminescence [12], photovoltaics [13,14], photochromism [15,16] and photodegradation [2,10]. The advanced oxidation process (AOP), an important property of semiconductors, has attracted the attention of researchers for the treatment of dye wastewater with enhanced degradation efficacy, physical and chemical properties and low toxicity. Owing to their filled valence band (VB) and empty conduction band (CB) in the ground state, semiconductors act as efficient photo-sensitizers in photocatalytic reactions. TiO_2_, one of the wide band gap inorganic semiconductors, is often used for photoelectric conversion and photocatalytic treatment for degrading a wide range of organic pollutants from water, since it is non-toxic, photostable, reusable and comparatively inexpensive [17,18,19]. Titanates, as compared to TiO_2_, demonstrate an enhanced intrinsic chemical reactivity, which is advantageous for designing complex titanate-based composite materials. Improved photocatalytic activity and chemical stability have been demonstrated by various titanates, namely CaTiO_3_ [20], SrTiO_3_ [2,21,22], BaTiO_3_ [23] and In_2_TiO_5_ [24]. Since the conduction band edge of strontium titanate is 200 mV more negative than TiO_2_, therefore it provides better energy assimilation for photocatalysis. SrTiO_3_ has been exploited extensively for the degradation of organic pollutants and water splitting under UV illumination [2]. However, the two foremost limiting aspects for the photocatalytic efficiency, namely low energy conversion due to rapid recombination of electron–hole (e^−^–h^+^) pairs and wide band gaps of semiconductors, restrict photo-absorption only within the UV region. Thus, numerous efforts have been made to enhance the photoresponse of semiconductors to the visible range to increase the photocatalytic properties [18,25].

Recently, conducting polymers with extend π-conjugated electron systems have been extensively explored for their electronic and optoelectronic properties. Due to their good environmental stability, high absorption coefficients in the visible spectrum and high electron–hole mobility, conducting polymers have attracted considerable attention of researchers. Owing to their significant electron-hole mobility properties, conducting polymers have been demonstrated to perform as stable photo-sensitizers for semiconductors, such as TiO_2_, ZnO, WO_3_, ZnS, *etc*. [26,27,28,29]. In multidisciplinary scientific research areas, conducting polymers, such as polypyrrole, polyaniline, polyphenylene, polythiophene and polyacetylene, have been exploited broadly, including for sensors [30], batteries [31], electronics and thermoelectric, electromagnetic, electro-luminescence and electromechanical applications [32,33,34]. Composite materials comprising conjugated polymers and wide band gap inorganic semiconductors have been widely investigated for exploring optical, photocatalytic and photoelectric conversion applications [35,36,37,38]. Polyaniline (PANI), due to its notable physical and chemical properties, such as thermal stability, high conductivity, easy preparation procedure, better processability, low cost and wide variety of applications [25], is one of the vastly investigated conducting polymers amid all of the conducting polymers. Additionally, PANI is an efficient electron donor and a good hole transporter upon visible light excitation. In the combined state with wide band gap semiconductors, PANI can transfer the electrons generated upon visible light irradiation to the conduction band of semiconductor, such as TiO_2,_ since the lowest unoccupied molecular orbital (LUMO) level of PANI is energetically higher than the conduction band (CB) edge of TiO_2_ [18]. As a consequence, a substantial amount of interfacial charge transfer occurs, and recombination of the electron-hole pair is considerably reduced, which could provide a significant photoresponse in the solar light range of the spectrum. This development will benefit the application of the photocatalysis under sunlight.

The present study centers on the degradation of methylene blue (MB) dye by polyaniline doped with SrTiO_3_ nanocubes synthesized via *in situ* oxidative polymerization. SrTiO_3_ nanocubes were synthesized using the simplistic hydrothermal technique and integrated into the polymer matrix during polymerization. PANI, due to its high electron mobility, enhanced the excitement of electrons under photo-illumination, and a facile method of synthesis was chosen as a base conducting polymer for designing efficient photocatalyst. Furthermore, SrTiO_3_ doping synergistically enhanced the generation of electrons and holes in combination with PANI to augment the degradability of MB. To evaluate the photocatalytic efficiency of synthesized polymeric composites, the photocatalytic treatment of MB in the aqueous phase for its degradation under visible light irradiation was chosen as a model reaction. Furthermore, the synthesized composite photocatalysts were characterized for morphological analysis, molecular structure and photo-responsive properties, and the effects of the weight ratio of polyaniline to SrTiO_3_ on the photocatalytic activities were studied.

## 2. Experimental Section

### 2.1. Materials

Aniline (Fluka, St. Louis, MO, USA, ≥99%) was distilled under reduced pressure and stored in the dark before use. Ammonium peroxydisulfate (APS, Merck, Kenilworth, NJ, USA, ≥99%), strontium hydroxide octahydrate (Sigma Aldrich, St. Louis, MO, USA, 95%), titanium(IV) oxide, anatase (Sigma Aldrich, St. Louis, MO, USA, 99.7%), sulfuric acid, H_2_SO_4_ (Sigma Aldrich, St. Louis, MO, USA, 98%), sodium hydroxide, NaOH (Sigma Aldrich, St. Louis, MO, USA, 98%), methanol (Merck, Kenilworth, NJ, USA, 99.9%) and acetone (Merck, Kenilworth, NJ, USA, 95%) were used as received without further purification. All of the reagents that were involved in the experiments were of analytical grade. Deionized water was used throughout the entire study.

### 2.2. Synthesis of SrTiO_3_ Nanocubes

A typical hydrothermal technique was utilized for the synthesis of SrTiO_3_ nanocubes. The calculated amount of strontium hydroxide octahydrate (1.4 g) was dissolved in 20 mL NaOH (3 M) under constant stirring. To this solution, titanium dioxide solution prepared by mixing 0.4 g of TiO_2_ in 20 mL NaOH (3 M) was added dropwise at a rate of one drop per second with vigorous stirring. After 30 min of stirring, 40 mL of the reaction mixture were transferred to a 100-mL Teflon-lined stainless steel autoclave and subjected to hydrothermal treatment at 130 °C for 72 h. The obtained precipitate of SrTiO_3_ nanocubes was then washed thoroughly with deionized water several times and dried in a vacuum oven at 100 millibars of pressure and 60 °C for 24 h.

### 2.3. Preparation of Polyaniline

Polyaniline was synthesized by the oxidative polymerization of distilled aniline that was dissolved in aqueous H_2_SO_4_ (0.5 M), using ammonium persulfate (APS) as an oxidant. The calculated amount of aniline (0.4 M, 40 mmol) was dissolved in 100 mL of an aqueous solution H_2_SO_4_, and APS (0.4 M, 40 mmol) was dissolved in 100 mL H_2_SO_4_ (0.5 M). The oxidant solution was then added slowly to the aniline solution with continuous stirring at 0–5 °C. The reaction mixture was stirred continuously for 3 h and kept in refrigerator overnight to complete the reaction. The reaction mixture was then filtered and washed with H_2_SO_4_ (0.1 M) until the filtrate became colorless and subsequently with deionized water until the filtrate became neutral. It was then washed with a mixture of acetone and methanol (1:1 ratio) to remove unreacted monomer and oligomers. The obtained polymer was dried in a vacuum oven at 100 millibars of pressure and 60 °C overnight. The green color of the obtained polymer indicated the formation of conductive polyaniline emeraldine salt.

### 2.4. Preparation of PANI-SrTiO_3_ Nanocomposite

SrTiO_3_ nanocube-doped nanocomposites were prepared with different wt% of SrTiO_3_ (250, 500 and 750 mg with respect to 0.4 M aniline, 40 mmol). The calculated amount of SrTiO_3_ nanocubes was dispersed in 5 mL of deionized water by sonication and added dropwise to aniline solution (0.4 M, 40 mmol) in H_2_SO_4_ with vigorous stirring. The resulting mixture was sonicated for a few minutes until it became uniform. The work-up procedure was the same as described in the previous section. The obtained nanocomposites were labelled as P-Sr250, P-Sr500 and P-Sr750, indicating 250, 500 and 750 mg of SrTiO_3_ nanocubes with respect to 0.4 M aniline, respectively. The dried samples were measured to calculate the yield and percentage of PANI loading in the nanocomposites (Table S1) according to the following Equation (1):

% PANI loading = [(*W*_1_ − *W*_2_)/*W*_0_] × 100 (1)

where *W*_1_ is the yield (g), *W*_2_ is the mass of SrTiO_3_ nanocubes (g) and *W*_0_ is the mass of aniline (g).

### 2.5. Characterization Techniques

The surface morphological and elemental analysis of the synthesized product was conducted using a JEOL JSM-7600F field emission scanning electron microscope operated at 10 kV. The size and shape of the obtained SrTiO_3_ nanocubes were studied using a JEOL JEM-2100F high-resolution transmission electron microscope. Thermal stability investigations were carried out using a Perkin Elmer TGA6 under an N_2_ atmosphere at a heating rate of 10 °C/min. Then, 10 mg of dried sample were loaded inside the alumina crucible, and the weight changes were monitored from 35–900 °C. X-ray diffraction (XRD) patterns were recorded using an Empyrean X-ray diffractometer from 2θ = 10°–90° using Cu Kα radiation (λ = 1.5418 Å) at a scan rate of 0.02 s^−1^. Surface area analyses were carried out by nitrogen adsorption/desorption isotherms at 77 K using a Micromeritics Tristar II ASAP 2020 system. The thermodynamic-based Barrett–Joyner–Halenda (BJH) method was used to determine the pore size data by using the desorption branch of the isotherm, whereas specific surface areas were measured using the Brunauer–Emmett–Teller (BET) method. Diffuse reflectance spectra were recorded with a UV–Vis spectrophotometer, Shimadzu Model UV-2550, against a BaSO_4_ white background. Fourier transform infrared (FTIR) spectra of the powdered samples were recorded using a Perkin Elmer RX1 FTIR ATR spectrometer in the range of 400–4000 cm^−1^ in spectral-grade KBr pellets.

### 2.6. Measurement of Photocatalytic Activities

The prepared samples were evaluated for photocatalytic activities by monitoring the degradation of methylene blue (MB) dye in the aqueous phase. In a typical experiment, 30 mg of the prepared nanocomposite powder were dispersed in 100 mL of an aqueous solution of MB with an initial concentration of 10 mg·L^−1^ in a quartz vessel. The adsorption–desorption equilibrium was achieved by stirring the mixture in a dark environment for 60 min. The photocatalytic degradation was then conducted by irradiating the above mixture using a 150-W Xenon Arc Lamp emitting solar light mimicking 1 Sun placed at a distance of 3 centimeters from solution. To maintain the uniform dispersion of photocatalyst particles, the mixture was stirred continuously. Then, 3 mL of the dye suspension were withdrawn at a regular time interval and centrifuged. The UV–Visible absorption spectra of the supernatant solution were analyzed using a UV–Visible spectrometer (Thermo Scientific Evolution) in 1-cm quartz cuvettes to monitor the characteristic absorption peak of MB.

## 3. Result and Discussion

### 3.1. Morphological Analysis of Nanocomposites

**Figure 1:**
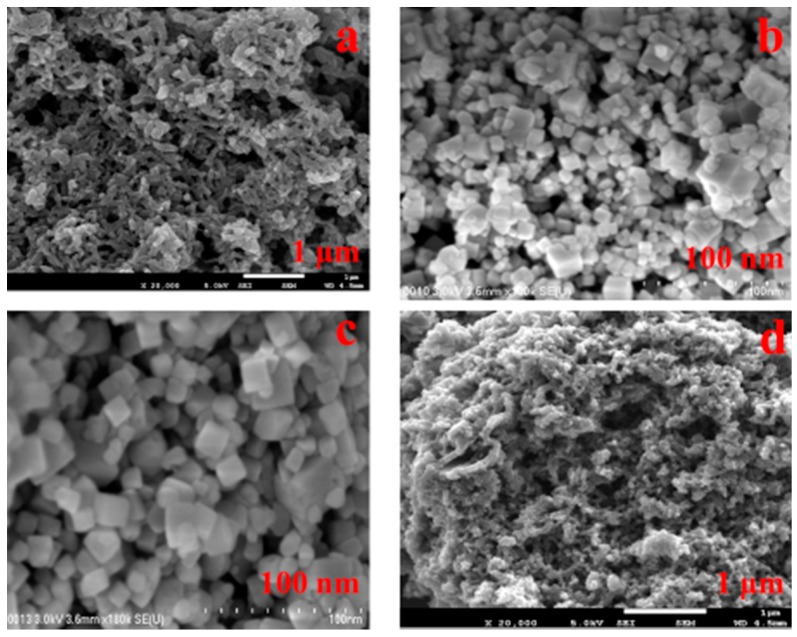
FESEM micrographs of (**a**) polyaniline (PANI); (**b**,**c**) SrTiO_3_ and (**d**) P-Sr500.

**Figure 2:**
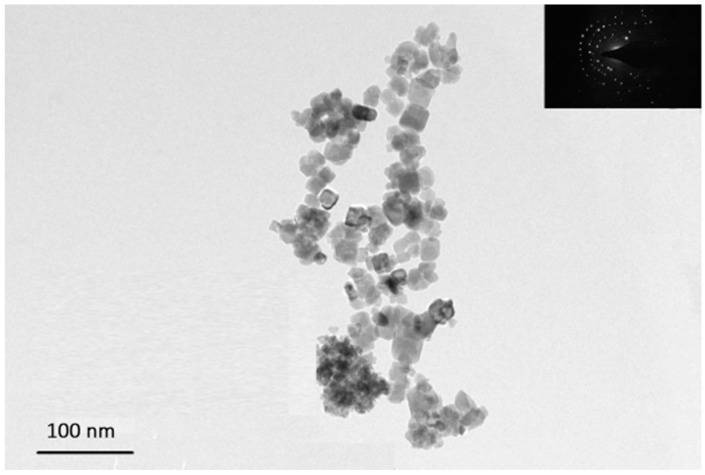
TEM image of SrTiO_3_ nanocubes. The inset highlights the SAED pattern of SrTiO_3_.

The structural characteristics and morphologies of PANI, SrTiO_3_ and SrTiO_3_ nanocube-doped polyaniline were examined by using FESEM. Figure 1a illustrates the morphology of PANI, which showed a flake-like structure as the characteristic structure of PANI. Figure 1b,c show the morphology of the strontium titanate nanocubes at different magnifications. These images clearly reveal the formation of cube-shaped strontium titanate nanoparticles with an approximately uniform particle size. Additionally, to confirm the formation of strontium titanate nanocubes, the synthesized nanoparticles were examined through TEM. Figure 2 demonstrates the TEM micrograph of strontium titanate with a selected area electron diffraction (SAED) pattern (inset), which clearly exhibits the presence of cube-shaped crystalline particles in the nano-range, confirming the formation of strontium titanate nanocubes. Figure 1d represents SrTiO_3_ nanocube-doped polyaniline nanocomposite material P-Sr500, revealing the formation of the granular polymeric network. As is evident from the Figure S1, the flake-like morphology of PANI was completely transformed into granular nanocrystalline polymeric nanocomposite material upon the addition of SrTiO_3_ into the polymeric matrix. Figure S2 shows the TEM image of P-Sr500, which indicates the presence of SrTiO_3_ nanoparticles in the polymer matrix of PANI.

**Figure 3:**
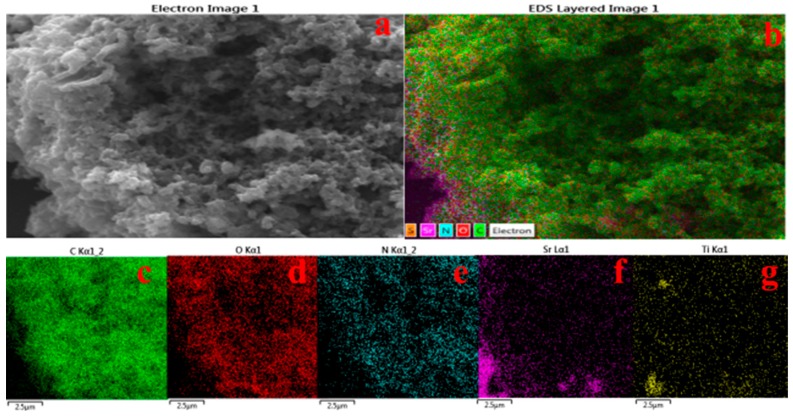
(**a**) FESEM image and (**b**) EDX elemental mapping of the P-Sr500 nanocomposite on a Si wafer for the following elements: (**c**) C; (**d**) O; (**e**) N; (**f**) Sr and (**g**) Ti.

Probably due to the low concentration of SrTiO_3_ nanocubes in nanocomposites (500 mg with respect to 0.4 M aniline), it is difficult to observe these nanoparticles in the FESEM images of the nanocomposite material, as these particles are embedded in the polymer matrix of polyaniline. Therefore, FESEM-EDX and FESEM-mapping may possibly prove to be a suitable technique to validate the presence of strontium titanate nanoparticles in the matrix of polyaniline. Figure 3 illustrates the mapping result of the nanocomposite with strontium titanate nanocubes (P-Sr500), which clearly reveals that strontium titanate (Figure 3f,g) is uniformly present in the nanocomposites along with carbon, nitrogen and oxygen. An elemental analysis (Figure S3) shows the presence of strontium and titanium, which further confirms the formation of strontium titanate nanocube-doped polyaniline nanocomposites.

### 3.2. Thermal Analysis

**Figure 4:**
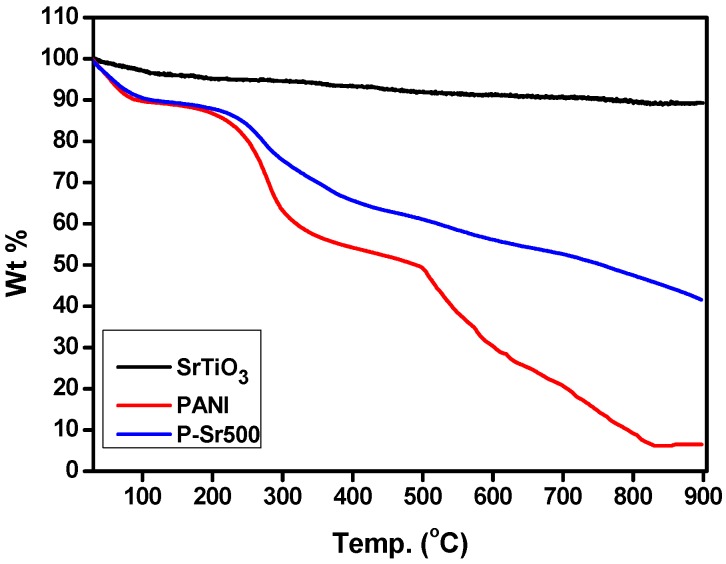
TGA thermogram analysis of PANI, SrTiO_3_ and P-Sr500 nanocomposite.

The thermal analysis of SrTiO_3_ nanocubes synthesized through the hydrothermal technique, PANI homopolymer and SrTiO_3_ nanocube-doped polyaniline nanocomposite, P-Sr500, was carried out using thermo gravimetric analysis performed under a nitrogen atmosphere by heating samples from 30–900 °C with a ramp rate of 10 °C/min. Figure 4 shows the TGA thermogram of the SrTiO_3_ nanocubes, PANI and P-Sr500. As is obvious from the TGA curve of SrTiO_3_, the first weight loss occurred between 50 and 150 °C, which may be attributed to the dehydration of the absorbed moisture. No prominent changes were observed in the TGA curve of SrTiO_3_, and total weight loss for a temperature range of 35–900 °C was found to be <10%. Three major weight losses were exhibited by the TGA thermogram of the PANI homopolymer. The first weight loss occurred from 40–120 °C, which may be due to the loss of adsorbed moisture, the decomposition of unreacted monomers and the decomposition of impurities. The second major weight loss is apparent from 120–260 °C, which may be accredited to the loss of the dopant (H_2_SO_4_), and the final weight loss appeared from 260–900 °C, which is possibly due to the decomposition of polymeric chains. As is evident from the graph, the thermal stability of SrTiO_3_ nanocube-doped polyaniline has improved to a greater extent as compared to bare homopolymer. Approximately 40% of P-Sr500 persisted as residue at the end of thermal analysis at 900 °C. Thus, the TGA analysis data clearly reveal that the thermal stability of P-Sr500 has been greatly enhanced due to the presence of thermally-stable pure SrTiO_3_ nanocubes in the nanocomposite material.

### 3.3. XRD Analysis

**Figure 5:**
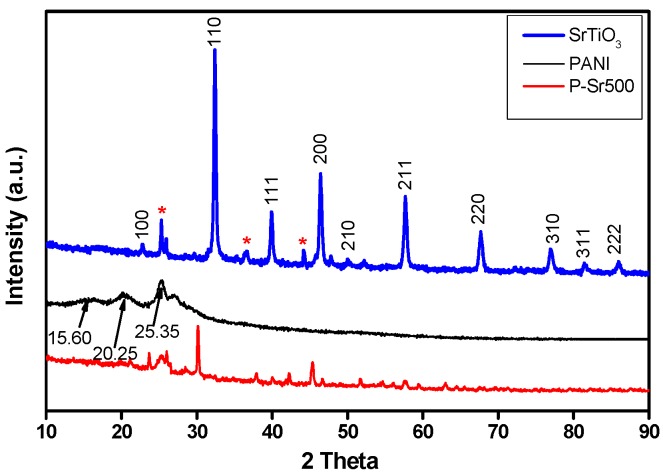
XRD patterns of PANI, SrTiO_3_ and P-Sr500 nanocomposite.

The typical room temperature XRD patterns of hydrothermally-synthesized SrTiO_3_ nanoparticles, PANI homopolymer and SrTiO_3_ nanocube-doped polyaniline nanocomposite are shown in Figure 5 and Figure S4. The XRD patterns of SrTiO_3_ nanocubes showed good crystallinity with diffraction peaks that correspond to the 100, 110, 111, 200, 210, 211, 220, 310, 311 and 222 planes of the cubic perovskite SrTiO_3_ structure, respectively. These peaks are characteristic of SrTiO_3_ and can be readily indexed as those of the cubic perovskite structure (space group: Pm3m) of SrTiO_3_ in accordance with JCPDS Card No. 35-0734. The peaks obtained for SrTiO_3_ were very sharp and well defined, which indicates the well-developed crystalline structure. The XRD patterns of SrTiO_3_ illustrated the presence of weak peaks at 2θ = 25.25°, 35.80° and 43.90° (marked with asterisk), which may be due to the presence of a minute amount of SrCO_3_ as contamination. The formation of a small quantity of SrCO_3_ is quiet obvious in the hydrothermal processing, which is due to the presence of CO_2_ from the air. CO_2_ dissolves in reaction medium as CO_3_^2−^ and reacts with Sr^2+^ ions, leading to the formation of SrCO_3_ during pretreatment and or the post-treatment [2,39]. The presence of SrCO_3_ will not cause any adverse effect on photocatalysis, and thus, it is negligible. For the PANI homopolymer, the diffraction appeared at 2θ = 15.6°, 20.25° and 25.35°, which are characteristic of PANI and indicate the polycrystalline structure of PANI [40]. The peaks at angles of 2θ = 20.25° and 25.35° correspond to the periodic repetition of benzenoid and quinoid rings in PANI chains [41]. As is apparent from Figure 5 and Figure S4, when strontium titanate nanocubes were doped into the polymer matrix of polyaniline, the broad and persistent peaks of PANI homopolymer were retarded to a greater extent. This diminishing of PANI crystalline peaks was enhanced with increasing the doping percentage of SrTiO_3_ and is possibly due to the fact that SrTiO_3_ nanocubes are acting as impurities that retard the growth of the PANI crystallite. Furthermore, the diffraction peaks of SrTiO_3_ in the nanocomposite become sharper and more apparent with increasing the doping percentage of SrTiO_3_ nanocubes. Thus, XRD analysis established successful synthesis of SrTiO_3_ and the formation of SrTiO_3_-doped polyaniline nanocomposites.

### 3.4. BET Analysis

**Figure 6:**
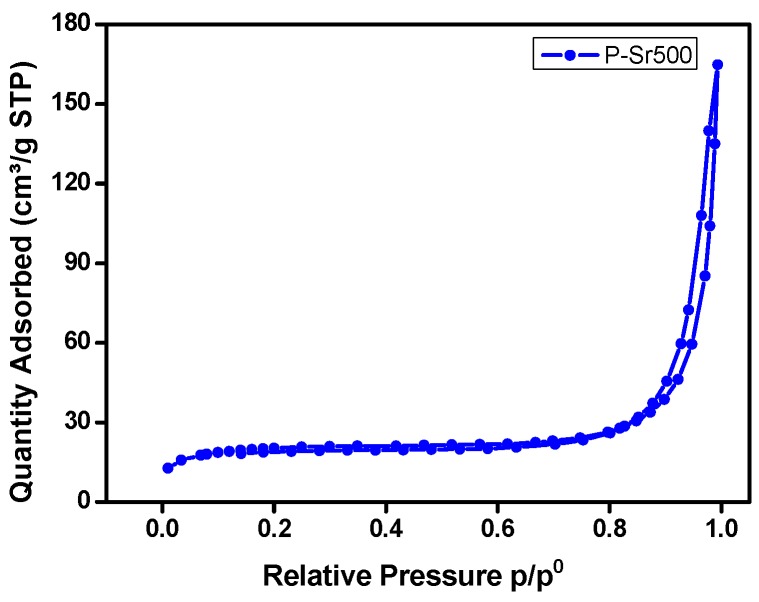
Nitrogen adsorption-desorption isotherms (BET) of P-Sr500 nanocomposite.

BET analysis was employed for investigating the specific surface area and porosity of SrTiO_3_ and SrTiO_3_ nanocube-doped nanocomposite by measuring the nitrogen adsorption-desorption isotherms. As shown in Figure 6 and Figure S5, the nitrogen adsorption-desorption isotherms of P-Sr500 and SrTiO_3_ demonstrated the characteristic of type-IV isotherms according to the International Union of Pure and Applied Chemistry (IUPAC) classification, which specifies their mesoporous nature [42,43]. The BET analyzer measurements showed the specific surface area for PANI homopolymer, SrTiO_3_ nanocubes and P-Sr500 nanocomposite to be 9.42, 9.20 and 16.50 m^2^·g^−1^, respectively. Figure S6 represents the BJH pore size distribution curve of P-Sr500 nanocomposite. As shown in Figure S6, the pore size distribution appears to be slightly inhomogeneous, and particles with a pore diameter of about 17 nm appear to be most abundant. The total volumes of pores for PANI homopolymer, SrTiO_3_ nanocubes and P-Sr500 nanocomposite were found to be 0.042, 0.026 and 0.06 cm^3^·g^−1^, respectively. Thus, P-Sr500 nanocomposite exhibits the high BET specific surface area and a large total volume of pores, as compared to PANI homopolymer and SrtiO_3_ nanocubes.

### 3.5. UV–Vis Analysis

**Figure 7:**
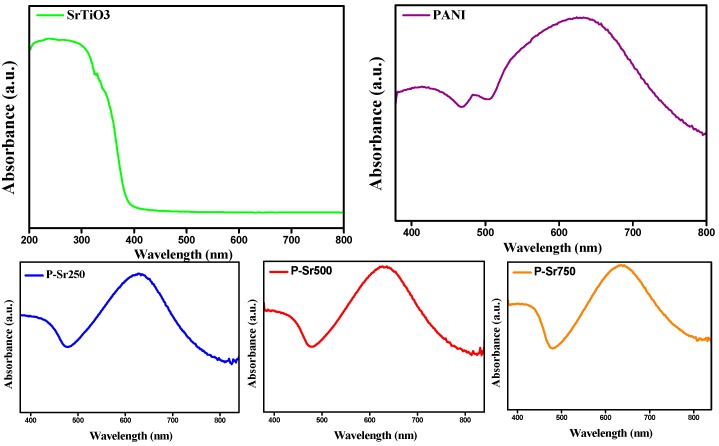
UV–Visible spectra of SrTiO_3_, PANI and different nanocomposites.

Figure 7 shows the UV–Vis diffuse reflectance spectrum for the SrTiO_3_ nanoparticles PANI homopolymer and SrTiO_3_ nanocube-doped polyaniline nanocomposites, namely P-Sr250, P-Sr500 and P-Sr750, respectively. As is evident from Figure 7a, SrTiO_3_ exhibits distinct broad absorption bands in the range of 200–398 nm, exhibiting the sharp absorption onset approximately at 398 nm, which may be attributed to the electronic transition from the valence band to the conduction band and is in agreement with the band gap edge absorption of SrTiO_3_ (3.2 eV) [44,45]. PANI homopolymer shows three characteristic absorption bands at 380−460, 470−500 and 505−780 nm, respectively, which are typical of the protonated form of PANI homopolymer [46]. The first absorption band can be assigned to the π–π* transition, the electron transition between the benzenoid segments. The second and third absorption bands can be assigned to the doping level and the π–π* transition of quinoid rings on the PANI chains due to the formation of polarons [10,47]. SrTiO_3_ nanocube-doped polyaniline nanocomposites show the optical absorption in the entire visible light region ranging from 480–800 nm, as shown by their diffuse adsorption spectrum. As these nanocomposites reveal absorption in the entire visible region, it is certain that they could function as effective light absorbers with enhanced photocatalytic properties.

### 3.6. FTIR Analysis

**Figure 8:**
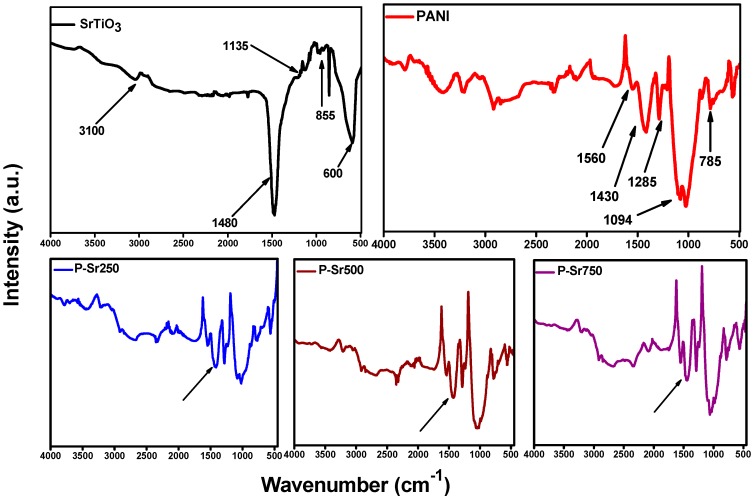
FTIR spectrum of SrtiO_3,_ PANI and different nanocomposites.

The FTIR spectra of PANI, strontium titanate nanocubes and SrTiO_3_ nanocube-doped polyaniline nanocomposite are shown in Figure 8. The FTIR spectrum of the PANI homopolymer exhibits the characteristic IR bands at 1560 and 1430 cm^−1^, which may be attributed to C–C stretching of quinoid and benzenoid rings in PANI, respectively. The peak at 1285 cm^−1^ could be assigned to C–N and C=N stretching in PANI. A characteristic broad IR band at 3425 cm^−1^ can be attributed to the N−H stretching mode in PANI. The occurrence of the benzenoid and quinoid units is the indication of the conductive emeraldine form of PANI. The IR spectrum of SrTiO_3_ exhibits a band around 3120 cm^−1^, which may be assigned to the O–H stretching modes in crystallization water [48].The IR peak at 1480 cm^−1^ corresponds to carboxylate group stretching modes [49], whereas the peak at 1135 cm^−1^ could be attributed to C=O stretching modes in COO–Sr. The absorption peaks at around 855 and 600 cm^−1^ are due to TiO_6_ octahedron bending and stretching vibration [50]. The FTIR spectrum of the SrTiO_3_ nanocube-doped PANI nanocomposites depicts the characteristic bands of the PANI homopolymer, but these bands are slightly shifted after the addition of SrTiO_3_ nanoparticles. The IR band at 1094 cm^−1^ in PANI was shifted to around 1040 cm^−1^ in SrTiO_3_^−^doped nanocomposites, while the intensity of the band at 1430 cm^−1^ (as indicated by the arrows in Figure 8) increases with the increase in the concentration of SrTiO_3_. This slight shifting of the band towards red could be attributed to some amount of weak van der Waals attraction between the polymer chain and SrTiO_3_. Hence, the FTIR studies are in good agreement with reported literature and clearly specify the formation of polyaniline, SrTiO_3_ and SrTiO_3_-doped polyaniline nanocomposites.

### 3.7. Photocatalytic Degradation of MB under Sunlight Irradiation

**Figure 9:**
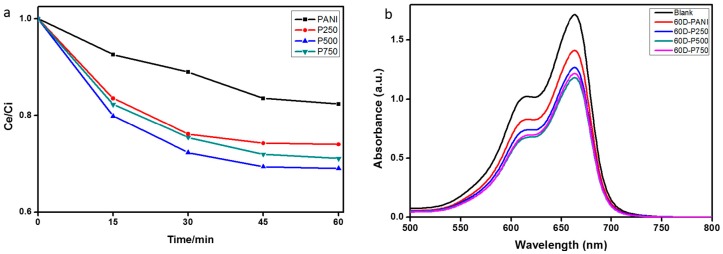
(**a**) Adsorption–desorption equilibrium rate of methylene blue (MB) under dark conditions *versus* time in the presence of various photocatalysts; (**b**) UV–Vis absorption spectra of MB aqueous solution at 60 min of dark adsorption–desorption equilibrium.

The photocatalytic aqueous phase decomposition of MB dye in the presence of the PANI homopolymer, P-Sr250, P-Sr500 and P-Sr750 nanocomposite catalysts was performed under natural sunlight irradiation at ambient temperature. In order to attain adsorption-desorption equilibrium, the adsorption of MB on the surface of the catalyst under dark conditions was monitored for 60 min via UV–Vis absorption spectra, as shown in Figure 9a,b. It is evident from Figure 9a that the adsorption of MB molecules on the surface of the catalyst augmented with time, and most of the catalyst surface was saturated with MB within 30 min. Approximately 18%, 26%, 31% and 29% of MB was adsorbed on the surface of PANI, P-Sr250, P-Sr500 and P-Sr750, respectively, after adsorption-desorption equilibrium was achieved. MB molecules can be adsorbed onto the surface of PANI with face-to-face coordination via π–π conjugation between MB and the aromatic regions of PANI, which increases the adsorptivity of MB on PANI, a prime requisition for enhanced photocatalytic activity. The adsorption of MB on the surface of photocatalyst is further enhanced by doping PANI with SrTiO_3_ nanocubes, which is probably owing to some molecular interaction of nanoparticles with MB. With increasing the percentage of SrTiO_3_ in the nanocomposites, the adsorption decreases slightly, which may be caused by the reduction in the effective surface area of the catalyst due to the agglomeration of SrTiO_3_ nanocubes at a high doping percentage.

**Figure 10:**
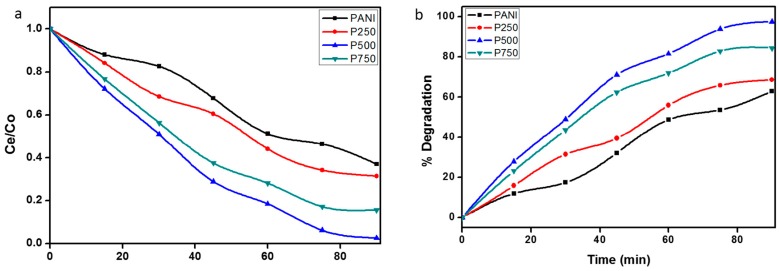
(**a**) Photodegradation rate of MB at different time intervals in the presence of various photocatalysts; (**b**) Percentage degradation of MB in the presence of various photocatalysts.

**Figure 11:**
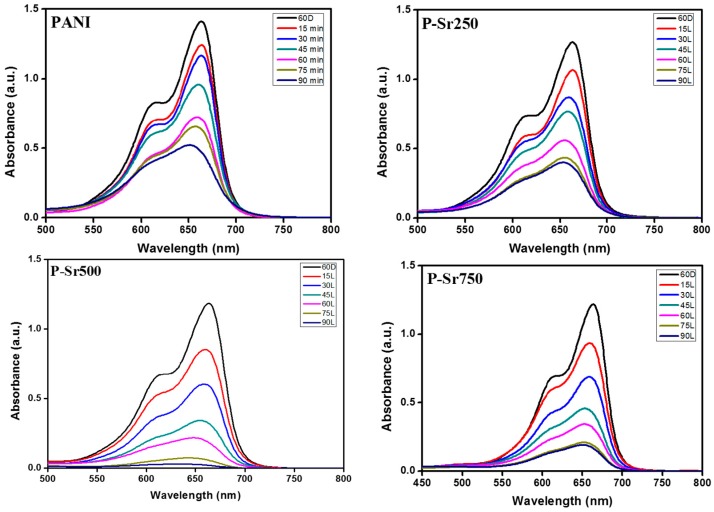
UV–Vis absorption spectra of MB aqueous solution at different times in the presence of different photocatalysts.

Figure 10a,b illustrates the rate of photodegradation of MB at different time intervals and the percentage of degradation of MB in the presence of various photocatalysts, which indicates that PANI exhibits low photocatalytic activity as compared to the photocatalytic activities of P-Sr250, P-Sr500 and P-Sr750 nanocomposite catalysts. The percentage of degradation, as evident from Figure 10a, follows the following trend: P-Sr500 > P-Sr750 > P-Sr250 > PANI. The UV–Vis adsorption spectra of the PANI and SrTiO_3_ nanocube-doped nanocomposites irradiated under solar light illumination for different time intervals are represented by Figure 11, which depicts the efficient degradation of MB by a photocatalytic phenomenon. As is evident from Figure 11, the intensity of the adsorption band decreases with the increase in irradiation time, and this decrease in intensity shows a different trend with each photocatalyst. The degradation percentage after 90 min of visible light illumination for PANI, P-Sr250, P-Sr500 and P-Sr750 nanocomposites was found to be 63%, 68%, 97% and 84%, respectively, which shows the enhanced photocatalytic activity of P-Sr500 over other photocatalysts. In PANI, degradation is 63% after 90 min, which could be due to high electron mobility in PANI chains through π–π* transitions under photo-illumination. The degree of decomposition enhances with the doping of SrTiO_3_ nanocubes in the conducting chains of PANI, which signifies the synergistic photocatalytic phenomenon. The photocatalytic degradation was enhanced greatly with increasing the concentration of SrTiO_3_ from P-Sr250 to P-Sr500. However, the photocatalytic degradation gradually decreased from P-Sr500 to Sr750, which might be owing to the reduction in the effective surface area of catalyst due to the agglomeration of SrTiO_3_ nanocubes at a high doping percentage, thereby predicting P-Sr500 to be an optimum photocatalyst composition.

MB dye can absorb light in the region of 500–700 nm, leading to the formation of singlet and triplet species by electronic transition and intersystem crossing and can undergo self-decomposition to a certain extent [51]. The singlet and triplet species owing to their higher energy and hyperactivity readily react with available oxygen, leading to the formation of superoxide, peroxide and hydroxyl radicals, also called advanced oxidation species (AOS). These AOS are extremely reactive species, which attack the dye, thereby degrading and mineralizing it effectively. However, the formation of AOS by MB alone is insignificant and needs to be enhanced with the aid of photoactive catalysts. Analogous to MB PANI, SrTiO_3_ and their hybrid nanocomposite photocatalysts can also generate AOS, which can actively degrade MB. PANI is an efficient electron donor, a good hole transporter upon visible light excitation and exhibits enhanced electron mobility upon photo-illumination. Conjugated conductive polyaniline itself possesses valence bands (highest occupied molecular orbital (HOMO)) and conduction bands (lowest unoccupied molecular orbital (LUMO)) similar to those of other conductors and semiconductors. Due to its characteristic π–π* transitions, PANI can excite electrons from its HOMO to LUMO upon photo-illumination by absorbing energy from photons. These characteristic transitions in PANI lead to the formation of electrons and holes in the HOMO and LUMO of PANI, respectively, which are further responsible for the generation of AOS, thereby degrading MB. However, the generation of AOS in PANI is low due to rapid electron–hole recombination, thus limiting the efficiency of PANI to be exploited as an effective photocatalyst. The photocatalytic efficiency of PANI was greatly improved via doping of SrTiO_3_ nanocubes into the conducting chains of PANI through the *in situ* polymerization process. SrTiO_3_, a wide band gap semiconductor (Eg = 3.2 eV), undergoes chemical bond interactions with the LUMO of PANI through its d-orbital conduction band upon photo-irradiation. These electronic interactions are responsible for the LUMO of PANI to come close to the conducting band of SrTiO_3_, thus leading to the injection of electrons from the LUMO of PANI to the CB of SrTiO_3_, thereby increasing the charge separation and limiting the electron–hole recombination. These electrons and holes reach the surface of the catalyst, where they react rapidly with oxygen and water molecules to form peroxides, superoxide and hydroxyl radicals, respectively, which effectively attack MB, causing its decomposition and mineralization. Hence, SrTiO_3_ works synergistically with PANI to greatly enhance the photocatalytic degradation of MB by expediting the formation of AOS. Figure S7 provides the schematic representation of the probable mechanism of MB degradation in the presence of SrTiO_3_ nanocube-doped nanocomposites.

### 3.8. Comparison of Photocatalytic Efficiencies

A comparative study of the photocatalytic efficiencies of the photocatalysts reported in the literature with the present catalyst is significant and is represented in Table 1. With a catalyst loading of 30 mg/100 mL at an initial dye concentration of 10 mg/L, the synthesized catalysts revealed a dye degradation efficiency of 97% with photo-illumination for 90 min. As is evident from Table 1, in previous studies for the degradation of MB with different times of photo-illumination, Autin *et al.* [27] reported 88% degradation; Eskizeybek *et al.* [52] reported 99% degradation; Kant *et al.* [53] reported 99.47% degradation; Handan *et al.* [47] reported 97% degradation; Dai *et al.* [54] reported 87% degradation; and Olad *et al.* [55] reported 82% degradation; using various photocatalysts. In the very latest investigations for the photocatalytic degradation of MB, the reported photodegradation was between 88% and 95% with the concentration ranging from 3–10 mg/L [10,29,56]. Thus, in this context, it is very well established that our catalyst offers higher degradation efficiencies.

**Table 1:** Comparative study of the photocatalytic efficiencies of the different photocatalysts.

Catalyst	Dye degraded	Conc. of dye (mg/L^−1^)	Amount of catalyst (mg/mL)	% Degradation	Degradation time (min)	Source of light	Reference
Chitosan/PANI/Co_3_O_4_	Methylene Blue	10	0.3	88	180	UV	[10]
Graphene/ZnS	Methylene Blue	10	0.2	95	180	UV	[29]
PANI/CdO	Methylene Blue	4.8	0.4	97	240	Sunlight	[47]
TiO_2_	Methylene Blue	5	–	88	120	LED	[27]
PANI/ZnO	Methylene Blue	3.2	0.4	99	300	Sunlight	[52]
PANI/FZNPs	Methylene Blue	3.2	0.25	99.47	300	Sunlight	[53]
AgBr/ZnO	Methylene Blue	10	1	87	240	LED	[54]
PANI/ZnO	Methylene Blue	10	300	82	60	Visible light	[55]
FeOOH-LDO	Methylene Blue	3	35	95	180	Visible light	[56]
PANI/SrTiO_3_	Methylene Blue	10	0.3	97	90	Visible light	This work

## 4. Conclusions

In summary, we present here a facile and distinctive route to synthesize nanostructured SrTiO_3_ nanocube-doped polyaniline nanocomposite through an *in situ* oxidative polymerization procedure. The synthesized nanomaterial exhibited enhanced specific surface area, as revealed by BET studies, uniform nanopore distribution, as depicted by FESEM and TEM, and good photocatalytic performance. SrTiO_3_ nanocubes incorporated into the matrix of PANI homopolymer demonstrated enhanced photocatalytic activity, indicating the synergistic phenomenon between conducting polymer and the semiconducting metal oxide. The concentration of the MB decreased up to 97% after 90 min of visible light irradiation. The proposed technique may be used for the synthesis of numerous nanocomposites materials, with other conducting polymers addressing the present-day issue of environmental pollution caused by various organic pollutants. Moreover, the enhanced photocatalytic activity in visible light is more economical compared to UV irradiation and may expedite generating industrial applications.

## Supplementary Materials

The following are available online at www.mdpi.com/2073-4360/8/2/27/s1. Table S1: Yield and % PANI loading in various nanocomposites; Figure S1: FESEM images of (a) PANI homopolymer and (b) P-Sr750; Figure S2: TEM image of P-Sr500; Figure S3: EDX spectrum of P-Sr500 nanocomposite; Figure S4: XRD patterns of P-Sr250 and P-Sr750; Figure S5: Nitrogen adsorption-desorption isotherms (BET) of SrTiO3 nanocomposite; Figure S6: BJH pore-size distribution of P-Sr500 nanocomposite; Figure S7: Proposed mechanism for the photocatalytic degradation of MB under UV irradiation.
