# Supplementary material for: SrTiO3 Nanocube-Doped Polyaniline Nanocomposites with Enhanced Photocatalytic Degradation of Methylene Blue under Visible Light
Source: Polymers (Basel). 2016 Feb 15;8(2):27. doi: 10.3390/polym8020027 (PMC6432585; doi:10.3390/polym8020027)
Supplement: Supplementary file 1 [file polymers-08-00027-s001.pdf]

Supplementary Materials: $SrTiO_3$ Nanocube-Doped Polyaniline Nanocomposites with Enhanced Photocatalytic Degradation of Methylene Blue under Visible Light

Syed Shahabuddin, Norazilawati Muhamad Sarih, Sharifah Mohamad and Juan Joon Ching

Table S1. Yield and % PANI loading in various nanocomposites.

Catalyst	Amount of $SrTiO_3$ (g)	Yield (g)	% PANI loading
PANI	–	3.512	–
P-Sr250	0.250	3.6846	92.2
P-Sr500	0.500	3.6701	85.1
P-Sr750	0.750	3.6370	77.5

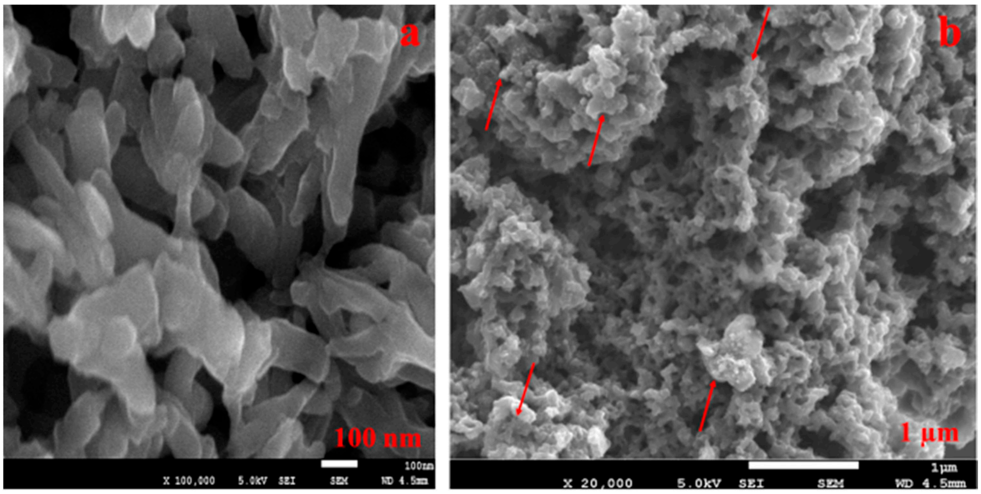

Figure S1. FESEM images of (**a**) PANI homopolymer and (**b**) P-Sr750 (Arrows represent the granular nanocomposite).

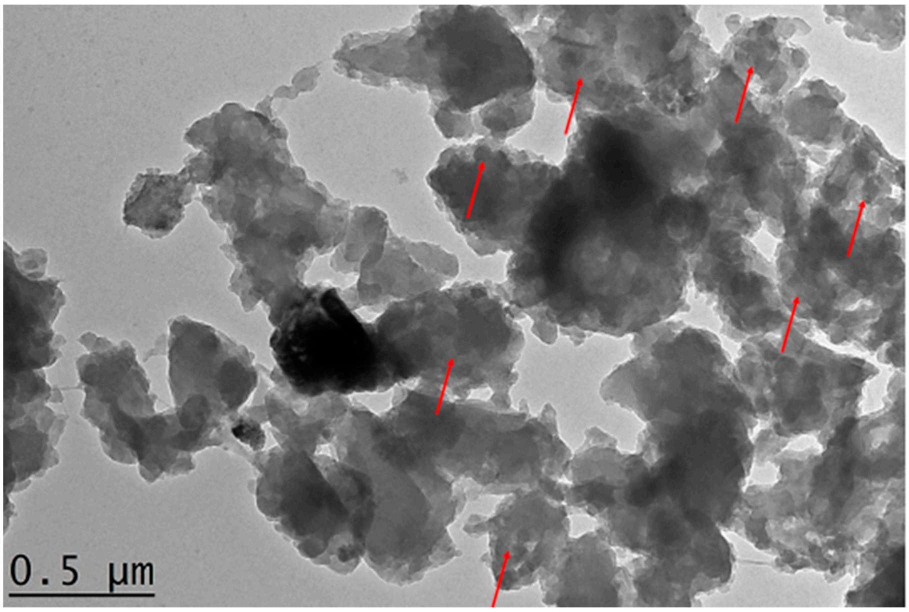

Figure S2. TEM image of P-Sr500 (Arrows specify the presence of $SrTiO_3$ nanocubes in polymer matrix).

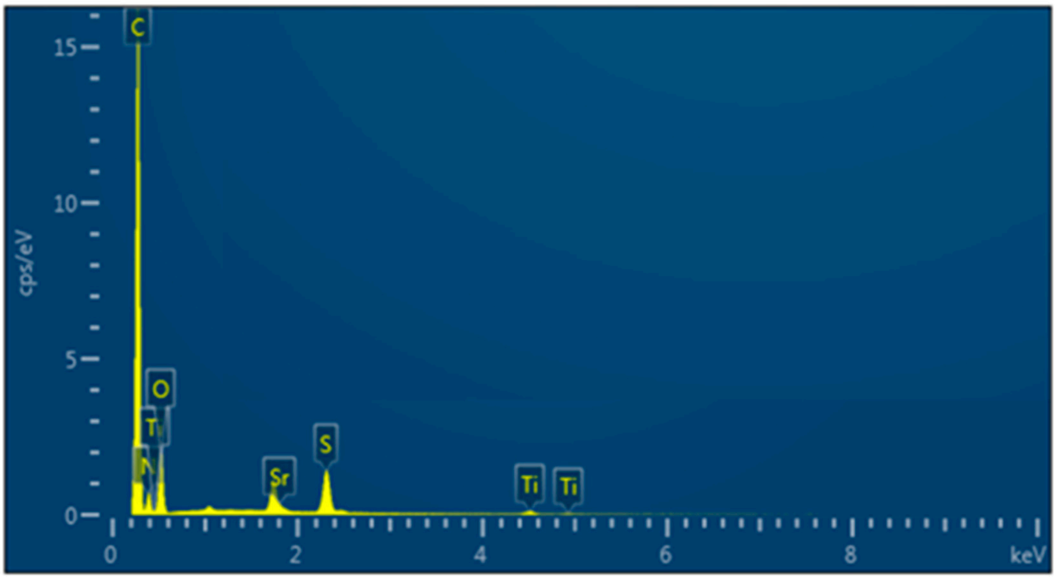

Figure S3. EDX spectrum of P-Sr500 nanocomposite.

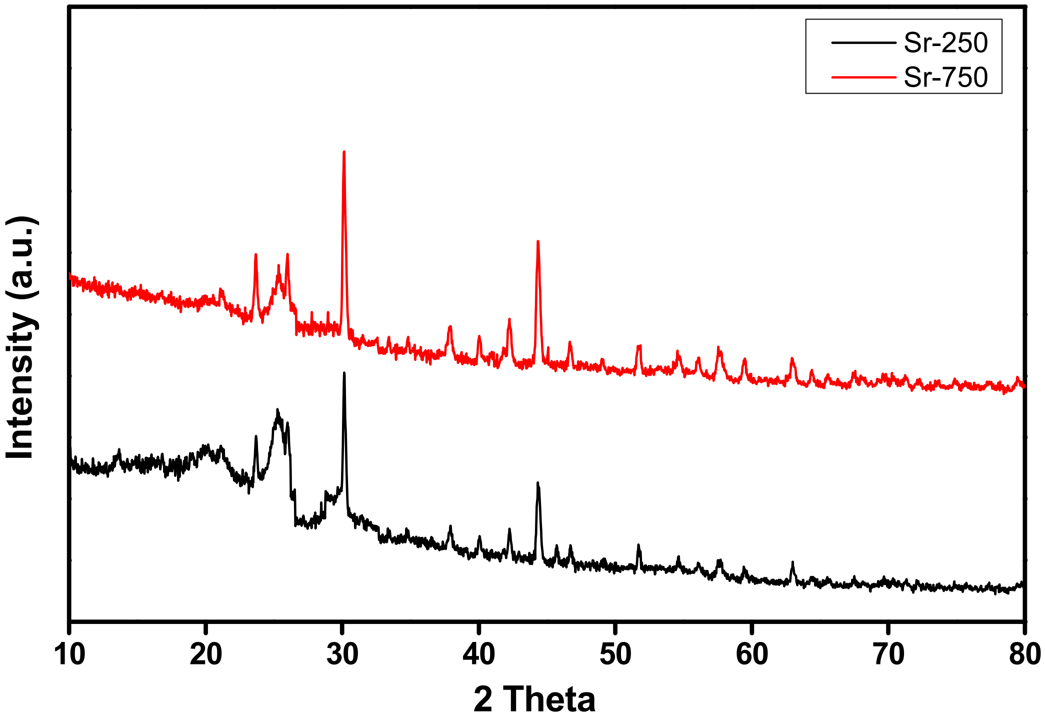

Figure S4. XRD patterns of P-Sr250 and P-Sr750.

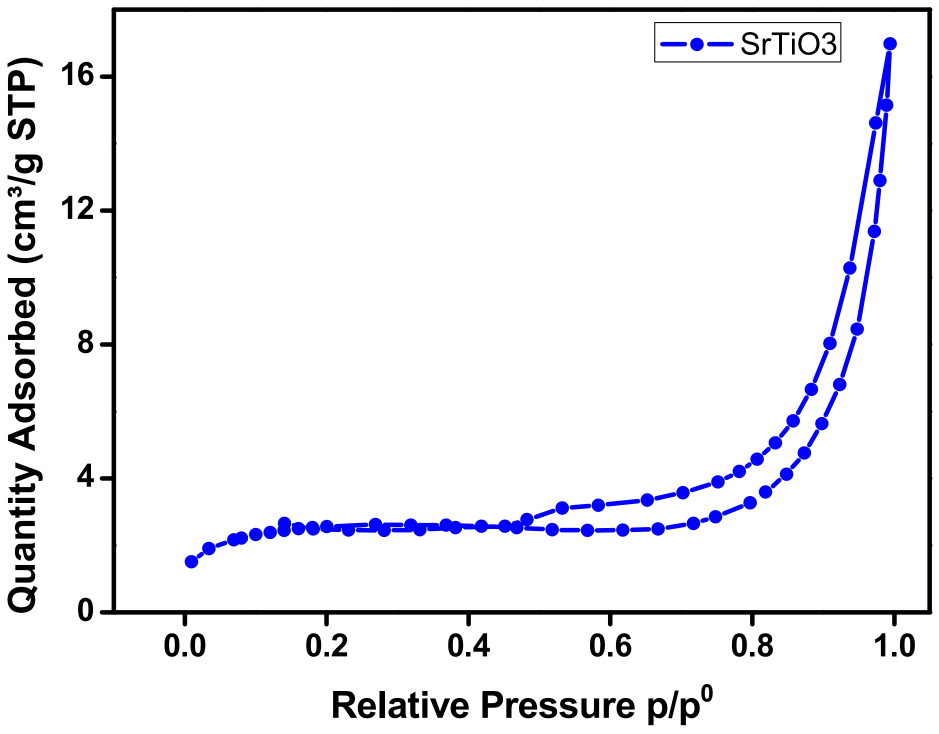

Figure S5. Nitrogen adsorption–desorption isotherms (BET) of SrTiO3 nanocomposite.

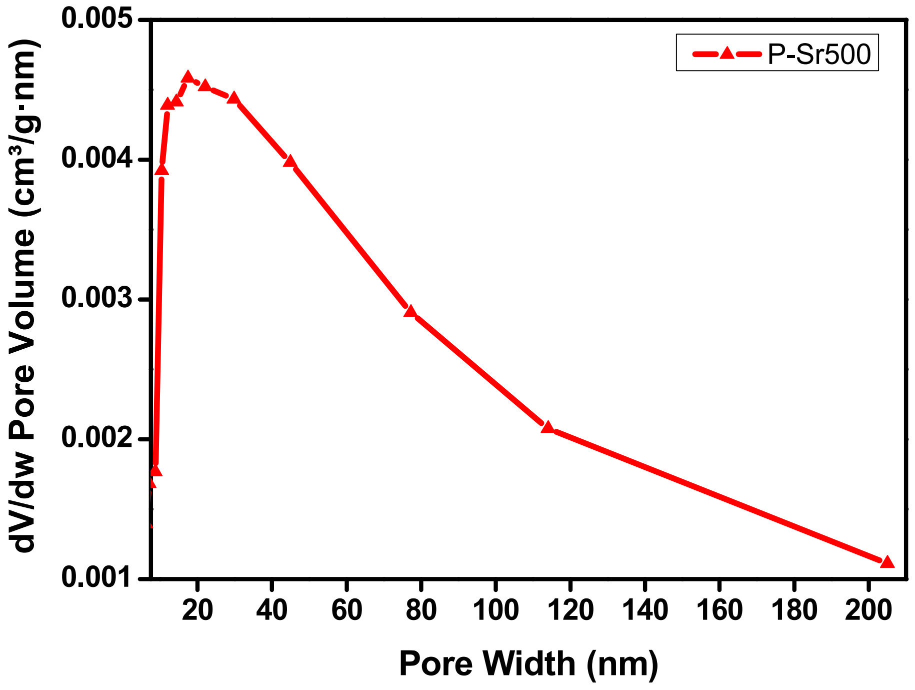

Figure S6. BJH pore-size distribution of P-Sr500 nanocomposite.

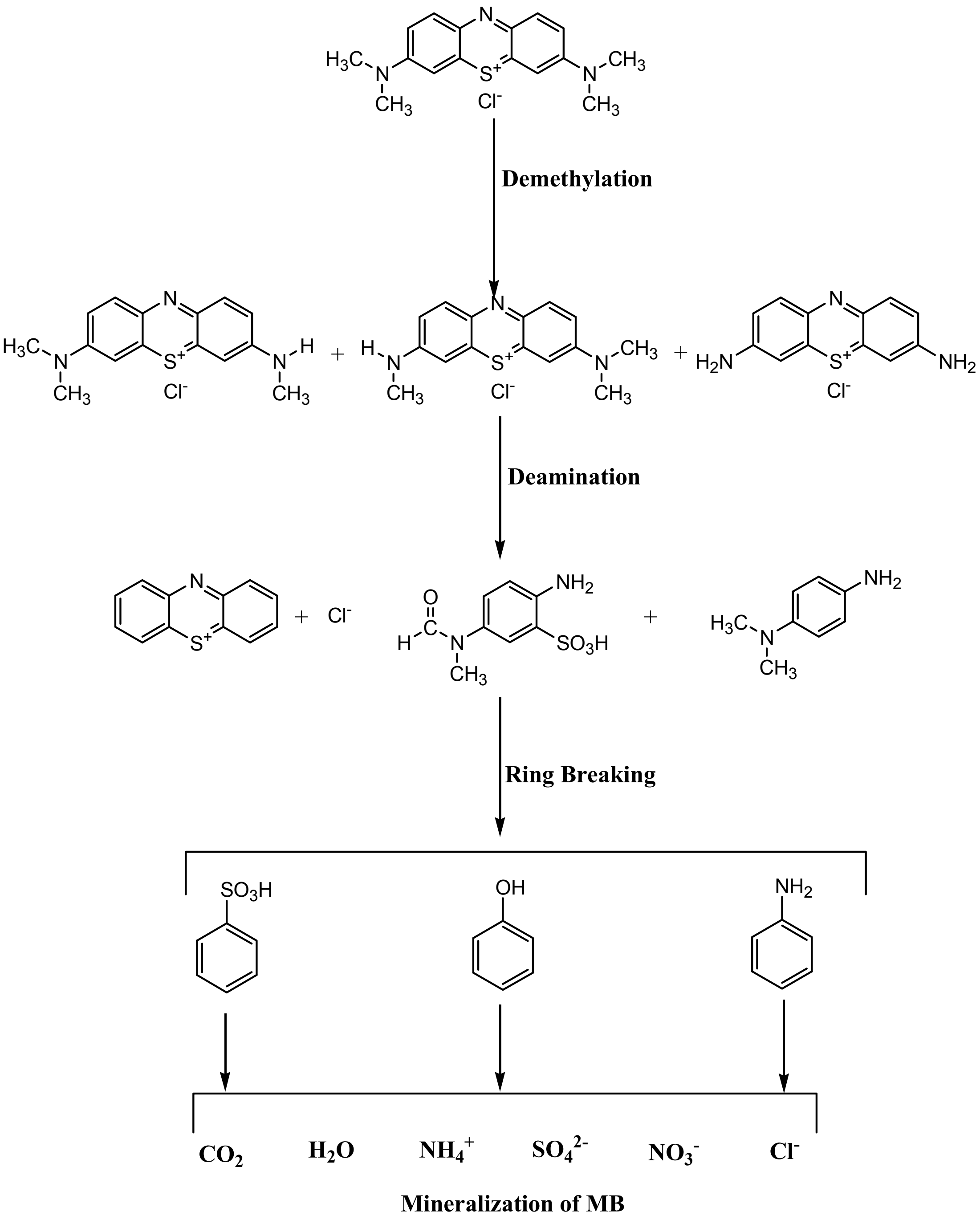

Figure S7. Proposed mechanism for the photocatalytic degradation of MB under UV irradiation.

© 2016 by the authors; licensee MDPI, Basel, Switzerland. This article is an open access article distributed under the terms and conditions of the Creative Commons by Attribution (CC-BY) license (http://creativecommons.org/licenses/by/4.0/).
